# Supplementary material for: Compressed Prostate Cancer Cells Decrease Osteoclast Activity While Enhancing Osteoblast Activity In Vitro
Source: Int J Mol Sci. 2023 Jan 1;24(1):759. doi: 10.3390/ijms24010759 (PMC9821660; doi:10.3390/ijms24010759)
Supplement: Supplementary file 1 [file ijms-24-00759-s001.zip › ijms-2085170-supplementary.pdf]

**Supplementary Table S1**. Number of undetectable samples and outliers after qPCR analyses, followed by the final sample size for each gene.

| *Gene (human)* | **Undetectable quantities** | **Identified outliers** | **Final sample size** |
|---|---|---|---|
| ***PTHrP*** | 0 | 4 | 16 |
| ***SYND1*** | 0 | 4 | 16 |
| ***Slug*** | 12 | 0 | 8 |
| ***Twist*** | 11 | 1 | 8 |
| ***Il6*** | 0 | 6 | 14 |
| ***TNF-α*** | 0 | 1 | 19 |
| ***WNT5a*** | 0 | 0 | 20 |
| ***PPARγ*** | 0 | 0 | 20 |
| ***TGF-β1*** | 0 | 3 | 17 |
| ***TGF-β2*** | 0 | 0 | 20 |
| ***GDF15*** | 6 | 0 | 14 |
| ***CYR61*** | 6 | 0 | 14 |
| ***Snail*** | 6 | 1 | 13 |
| ***KI67*** | 6 | 0 | 14 |
| ***VMN*** | 6 | 0 | 14 |
| ***CDH1*** | 6 | 0 | 14 |
